# Insights into Taxol® biosynthesis by endophytic fungi

**DOI:** 10.1007/s00253-023-12713-y

**Published:** 2023-08-22

**Authors:** Kamalraj Subban, Frank Kempken

**Affiliations:** Department of Genetics & Molecular Biology in Botany, Botanical Institute and Botanical Garden, Christian-Albrecht University of Kiel, Olshausenstraße 40, 24098 Kiel, Germany

**Keywords:** Taxol®, Endophytic fungi, Fungal Taxol biosynthesis, Taxol biosynthetic pathways, Orthologous genes, Unigenes

## Abstract

**Abstract:**

There have been two hundred reports that endophytic fungi produce Taxol®, but its production yield is often rather low. Although considerable efforts have been made to increase Taxol/taxanes production in fungi by manipulating cocultures, mutagenesis, genome shuffles, and gene overexpression, little is known about the molecular signatures of Taxol biosynthesis and its regulation. It is known that some fungi have orthologs of the Taxol biosynthetic pathway, but the overall architecture of this pathway is unknown. A biosynthetic putative gene homology approach, combined with genomics and transcriptomics analysis, revealed that a few genes for metabolite residues may be located on dispensable chromosomes. This review explores a number of crucial topics (i) finding biosynthetic pathway genes using precursors, elicitors, and inhibitors; (ii) orthologs of the Taxol biosynthetic pathway for rate-limiting genes/enzymes; and (iii) genomics and transcriptomics can be used to accurately predict biosynthetic putative genes and regulators. This provides promising targets for future genetic engineering approaches to produce fungal Taxol and precursors.

**Key points:**

*• A recent trend in predicting Taxol biosynthetic pathway from endophytic fungi.*

*• Understanding the Taxol biosynthetic pathway and related enzymes in fungi.*

*• The genetic evidence and formation of taxane from endophytic fungi.*

## Introduction

A bestseller anticancer drug, Taxol® (paclitaxel), contains an unusual oxytane ring with a tricyclic core. Globally, there is a high demand for Taxol, but there is a low yield from *Taxus* species (Suffness and Wall 1995; Kusari et al. 2014) and this led to environmental concerns. Currently, Taxol is obtained from semi-synthesis, plant cell culture, and wild *Taxus* plant resources that increase the cost effectiveness of Taxol (Frense 2007). Improving taxane production from *Taxus* plant through genetic engineering still faces challenges such as gene transformation, regulation of transcription factor, and overexpression of rate-limiting genes (Perez-Matas et al. 2023). In addition, there have been efforts to produce taxanes in bacteria and yeasts by heterologous expression (Perez-Matas et al. 2023). However, the successful transfer of the entire taxane biosynthetic pathway to these organisms has not been achieved yet. According to Nazhand et al. (2020) and Mohamed et al. (2023) Taxol and its derivatives are worth over $1 billion per year.

First reported paclitaxel producing endophytic fungus *Taxomyces andreanae* was described in 1993 (Stierle et al. 1993), highlighting the potential for fermenting and genetically manipulating fungi to produce paclitaxel. Endophytic fungi may offer several advantages, including the ability to grow quickly in simple culture media, the ability to cultivate in large fermenters, and the ability to produce Taxol easily. Taxol is produced by approximately 200 endophytic fungi associated with both *Taxus* species and non-*Taxus* species (McElroy and Jennewein 2018), but as a result of a lack of information about the Taxol biosynthetic pathway, their industrial utilization has remained elusive.

A terpenoid is synthesized first, followed by a phenylpropanoid pathway in which a benzoylphenylisoserine moiety is attached to C13 of the baccatin III skeleton (Zhou et al. 2019). This pathway is based on the taxadiene synthase (TS) gene, which catalyzes the cyclization of geranylgeranyl diphosphate (GGPP) into taxadiene in endophytic fungi. Following this are hydroxylase genes taxane-10β-hydroxylase (T10βOH) and taxadiene-13α-hydroxylase (T13αOH), as well as different acyltransferases taxadiene-5α-ol-O-acetyl transferase (TAT), 10-deacetylbaccatin III-10-O-acetyl transferase (DBAT) and baccatin III: 3-amino, 13-phenylpropanoyltransferase (BAPT). In the final step, the β-phenylalanoyl side chain is formed by BAPT (El-Sayed et al. 2019a, b; Kusari et al. 2014; Vasundhara et al. 2016). However, it is unknown what role orthologous genes from endophytic fungi play.

**Fig. 1:**
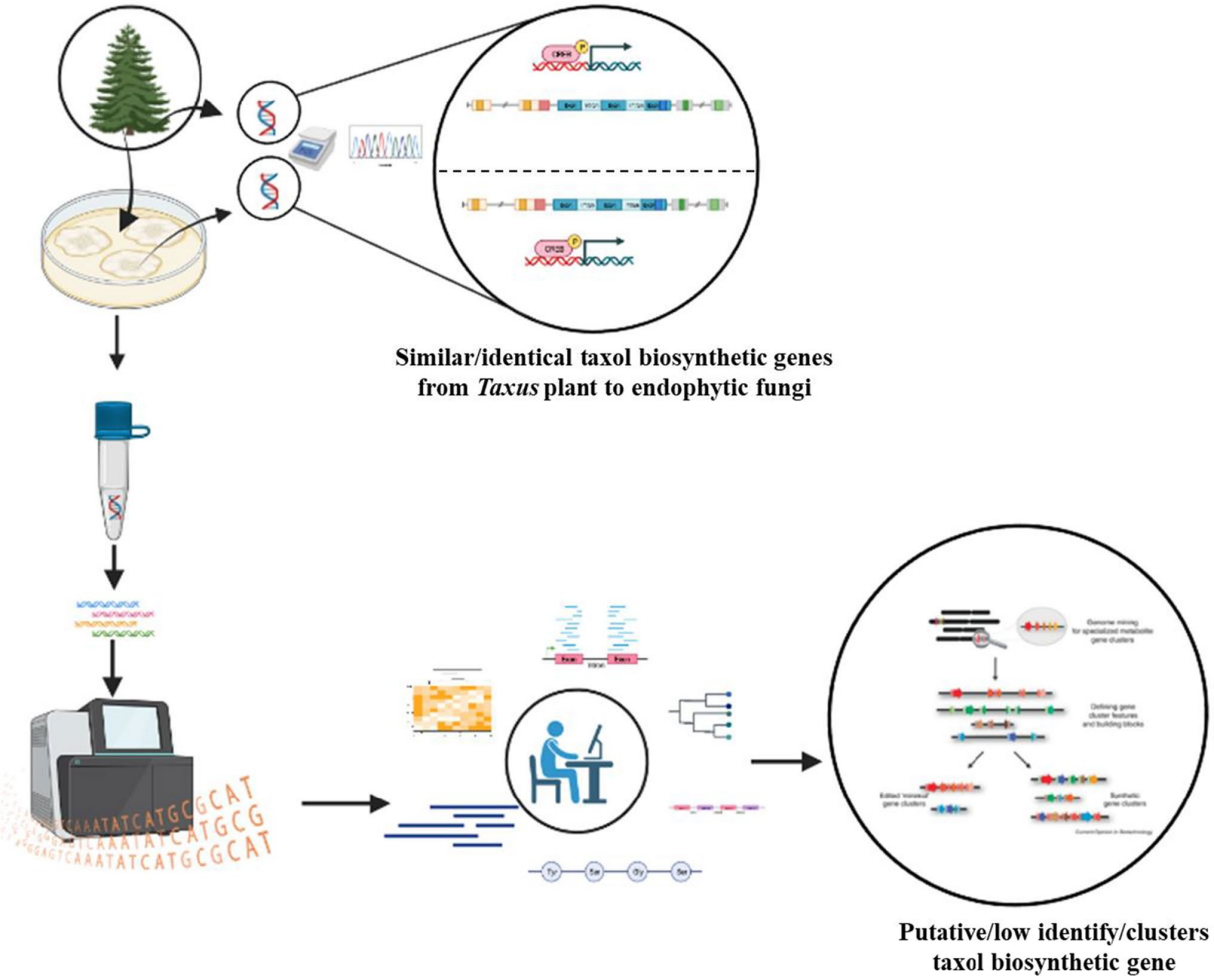
Finding the orthologs Taxol biosynthetic genes by PCR-based approaches and predicting putative Taxol biosynthetic genes by next-generation sequence based from endophytic fungi. (concept image made by BioRender scientific illustration software)

Endophytic fungi may have an independent paclitaxel biosynthesis pathway, but until now this has not been revealed by whole genome sequencing (Fig. 1). Taxol biosynthetic genes have been characterized at the transcript level, but there is no detailed sequence information (El-Sayed et al. 2018, 2019a, b). Only recently, homologous unigenes (a unique transcript that is transcribed from a genome), TS, cytochrome P450 taxadiene-5α-hydroxylase (T5αH), taxane 13-alpha-hydroxylase (T13αH), and taxane-2α-O-benzoyl transferase (TBT) were found in Taxol producing fungi by genomic and transcriptomic analysis (Yang et al. 2014; Miao et al. 2018; Qiao et al. 2020), but further verification at the enzymatic level is still missing. In contrast, there has been a significant pause in development efforts around the Taxol biosynthetic pathway in endophytic fungi over the past 3 decades. Thus, the purpose of this review is to describe the current status of fungi that produce paclitaxel, including those recently discovered. It will be discussed the advances due to whole genome sequencing, and Taxol biosynthetic pathway genes found in *Taxus* plants, along with ongoing efforts to genetically engineer Taxol production.

## Origin of fungal Taxol biosynthetic pathway

The exact evolutionary origin of the Taxol biosynthetic pathway in fungi is still unknown (McElroy and Jennewein 2018). There is a possibility that fungi acquired the ability to synthesize Taxol through horizontal gene transfer (HGT) from *Taxus* plants to endophytic fungi (Kusari et al. 2014). It is fascinating why so many fungi have orthologs of Taxol biosynthesis genes. It is unknown whether HGT plays a role in the transfer of biosynthetic genes from the host to endophytes (El-Sayed et al. 2019a, b, 2020; Chakravarthi et al. 2020; Guo et al. 2006). Plants and fungi have profoundly different promoter and intronic sequences. However, further studies are needed to explain how transferring complete and complex biosynthetic pathways allows for Taxol production among the endophytic fungi, even though the transfer of Taxol biosynthesis genes and transcription factors is still in their infancy.

Numerous reports demonstrate that the Taxol-producing fungal genes are 90% identical to the corresponding *Taxus* plant genes (Zhang et al. 2019a, b; Staniek et al. 2009; Chakravarthi et al. 2020). This raises the question of whether the Taxol biosynthetic pathway in fungi and *Taxus* species is the same. Yuan et al. (2006) speculated that the Taxol biosynthetic pathway might be conserved between *Taxus* plants and endophytic fungi (McElroy and Jennewein 2018; Sagita et al. 2021). In order to explain this surprising finding, two main hypotheses have been proposed: (i) HGT or (ii) convergent evolution (Soliman and Tang 2014), a topic that is still widely debated. Biosynthesis pathways of secondary metabolites (like gibberellins) in plants and fungi are examples of convergent evolution. There might be convergent or parallel evolution at work (Yang et al. 2014; Hedden et al. 2001). This, however, would not explain why endophytic fungi associated with plants that do not produce *Taxus* have Taxol biosynthesis genes.

## Taxol biosynthesis

Genes associated with the Taxol synthase pathway in *Taxus* spp. have not been extensively studied in Taxol-producing endophytic fungi. To understand the mechanism behind fungal Taxol biosynthesis, many more studies are required. It was possible to clone Taxol biosynthesis genes from several different Taxol-producing endophytic fungi by polymerase chain reaction (PCR)-based screening using primers designed from *Taxus* plant gene sequences (Flores-Bustamante et al. 2010; Hao et al. 2013; Staniek et al. 2009).

A typical Taxol biosynthetic pathway consists of three steps, namely, the diterpene precursor biosynthesis of taxadiene by involving more carbocycles, the biosynthesis of baccatin III by involving more hydroxylase and acetylase enzymes, and the formation of Taxol side chains by phenylation. A more detailed explanation of these stages is provided for endophytic fungi that produce Taxol.

### Taxadiene biosynthesis

There are almost exclusively *Taxus* species that produce taxadiene. Taxol-diene-cyclase can convert GGPP into taxa-4 (5) (12)-diene, the backbone of Taxol-tricyclic-diterpene. The coding sequence for TS, the enzyme involved in the formation of the taxane skeleton, was identified by PCR amplification in many but not all Taxol producing fungi (Zhang et al. 2008; Miao et al. 2009; Staniek et al. 2009; Garyali et al. 2013; Choi et al. 2016; Andrade et al. 2018; Abdollah et al. 2017; Xiong et al. 2013; Mirjalili et al. 2012; Das et al. 2017a; Kumaran et al. 2011), but Taxol production was also reported in the presence and absence of TS in non-*Taxus* plant-associated fungi (Sah et al. 2017; Kumaran et al. 2011). A fungal TS gene sequence of 632 bp was reported in *Mucor rouxianus* isolated from *T. chinensis* (Miao et al. 2009) and exhibited 98% identity to *T. brevifolia.* Soliman et al. (2013) confirmed that western blot analysis with a polyclonal anti-TS antibody revealed the appearance of a protein of similar size to plant TS in the endophytic fungi, suggesting the fungus is capable of producing Taxol. Intriguingly, *Taxus* plant-associated fungi only shared genes with *Taxus* plants.

**Fig. 2:**
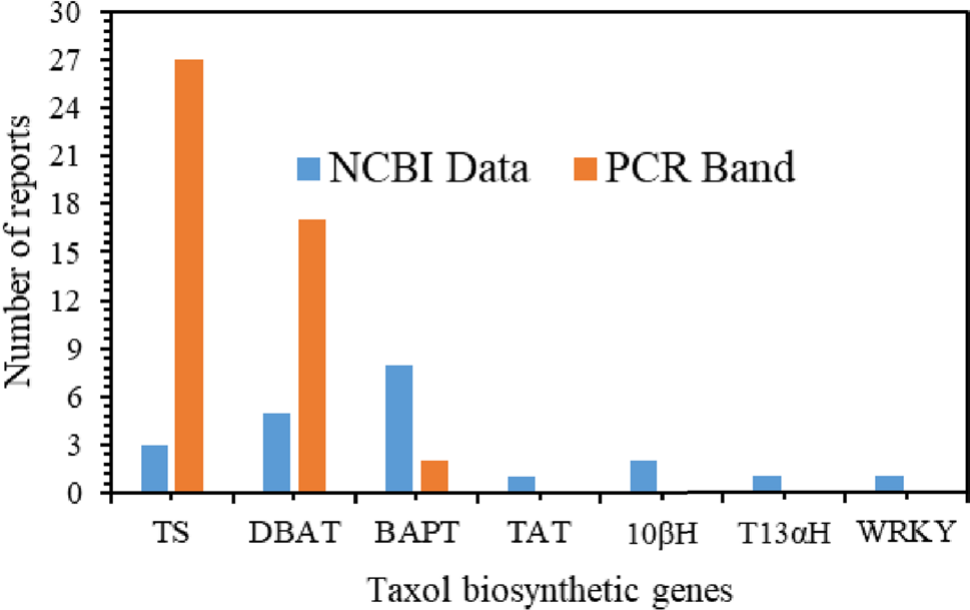
Orthologs of Taxol biosynthetic pathway genes and transcription factor reported from fungi

Few studies have examined the expression of the TS gene (El-Sayed et al. 2019a, b, 2020) in fungal strains isolated from *Podocarpus* (a non-*Taxus* plant), but none provided sequence data. Moreover, a report found that the Taxol biosynthetic gene sequence from endophytic fungus (HM113487) had 99% identity with its *Taxus* plant counterpart (Fig. 2). Thus, McElroy and Jennewein (2018) suggested that these sequences and PCR products are the result of host DNA contamination. Consequently, the fungal TS genes have not yet been fully sequenced.

### Biosynthesis of 10-de acetyl baccatin III and baccatin III

In addition to its unique structure, Taxa-4(5),11(12)-diene is composed of a taxane core molecule that is altered with alcohol functions, carbonyls, and cyclic ethers to produce 10-deacetylbaccatin III (Jennewein et al. 2001). Several hydroxylase and acetyltransferase genes from *Taxus* species have been identified in the past. Some of these have been cloned and characterized, including 5α-hydroxylase, 5α-ol-O-acetyltransferase, 10β-hydroxylase (Schoendorf et al. 2009), 13α-hydroxylase (Jennewein et al. 2001), and 14-hydroxylase (Jennewein et al. 2003). It is still necessary to clone and characterize some of these genes in the *Taxus* plant (McElroy and Jennewein 2018). It was reported that an ortholog Taxol biosynthetic gene open reading frame (ORF) of (AY960682) TAT was found in an *Ozonium* BT2 fungal isolate, and a complete coding sequence was derived from the *Ozonium* P450 taxane 10-beta-hydroxylase gene (AY907826) (Guo et al. 2006). Recently, partial cDNAs (EF626531) of the T13αH were shown in *Fusarium solani* (Chakravarthi et al. 2020), which may be another indication of HGT from plants to fungi (Kusari et al. 2014). Walker and Croteau (2000a) describe the DBAT as a rate-limiting enzyme in Taxol biosynthesis. By catalyzing the conversion of 10-deactylbaccatin III to baccatin III, the DBAT plays an important role in developing Taxol (Wakler and Croteau 2000a). By cytochrome P450-mediated oxygenations, 10-deacetylbaccatin III and baccatin III play a major role in Taxol biosynthesis (Jennewein et al. 2001). Sah et al. (2017) cloned and sequenced a DBAT and WRKYGQK heptapeptide 1 (WRKY1) transcription factor from *Lasiodiplodia theobromae* fungus. Moreover, *Lasiodiplodia theobromae* DBAT (*Lt*DBAT) recombinant enzymes showed that 10-deactylbaccatin III could be converted into baccatin III (Sah et al. 2019, 2020). A further indication of a fungal DBAT gene is shown in Fig. 2.

### Biosynthesis of the Taxol side chain

In Taxol biosynthesis, phenylalanine is a key precursor for side chains. In this process, α-phenylalanine is transformed into β-phenylalanine, which is then transformed into phenylisoserine after hydroxylation. The C-13 phenylpropanoid side chain-CoA acyltransferase (BAPT) is a rate-limiting enzyme that is used as a molecular marker to screen for Taxol-producing endophytic fungi. PCR-based approaches were used to obtain BAPT genes and sequences for a large number of fungi (Fig. 2). Plant-based BAPT primers were used to identify a PCR product from the Taxol-producing fungus *Fusarium redolens*. It exhibited 94% identity with the gene of a *Taxus* plant (Garyali et al. 2013). Furthermore, BAPT genes from *F. avenaceum* and *F. tricinctum* were found to be highly homologous with those in *Taxus* plants (KF010843.1; KF010842.1). Interestingly, phenylalanine aminomutase (PAM) from *Cryptosporiopsis tarraconensis* was identified as a bottleneck and rate-limiting enzyme (Ballakuti and Ghanati 2023).

We found orthologs of TS, GGPP, WRKY, ethylene responsive factor (ERF), and apetala 2 (AP2)-related genes from endophytic fungi associated with *Taxus* plants, but not in fungi of unrelated species (unpublished data). We are working on predicting the biosynthesis pathway of Taxol.

The major question is, why do endophytic fungi possess orthologous genes for the Taxol biosynthesis pathway? How do endophytic fungi function metabolically and physiologically? Why do most endophytic fungi produce Taxol but not non-endophytic fungi? Future research will certainly focus on the biological functions and possible roles of plant-fungal interactions, fungal-fungal interactions, fungal-bacteria interactions, and fungal-insect interactions to know the answer of these questions.

## Prediction of putative fungal Taxol biosynthetic genes through next-generation sequencing

Despite their ability to produce Taxol, fungi endophytes are not extensively studied for their biosynthetic pathway genes or enzymes. The biosynthesis of Taxol involves two metabolic pathways, namely, the terpenoid pathway and the phenylisoserine side chain pathway (Croteau et al. 2006; Steele et al. 2005). In order to fully understand the biosynthesis of Taxol in the *Taxus* plant, further research is required (McElroy and Jennewein 2018). Endophytic fungus EF0021, which is associated with *Taxus* spp., does not have the genes for the Taxol pathway in its genome (Heinig et al. 2013). According to Yang et al. (2014), genome sequencing of *Penicillium aurantiogriseum* NRRL 62431 endophyte isolated from hazel revealed that Taxol biosynthesis evolved independently. In contrast, a conserved ORF of DBAT has been discovered in *C. cladosporioides* MD2 which has 97–99% identity with *Taxus* species (Zhang et al. 2009b; Flores-Bustamante et al. 2010). A recent transcriptomic approach with *C. cladosporioides* MD2 showed no sequences with similarity to *Taxus* plant DBAT, BAPT, and 3′-N-debenzoyl-2′-deoxytaxol-N-benzoyltransferase (DBTNBT) genes (Miao et al. 2018). The authors noted the existence of DBAT BAPT, DBTNBT genes in the fungal genome; however, they appeared to be silenced due to continuous subculture. Hence, these genes could not be annotated or detected by transcriptome analysis.

**Fig. 3:**
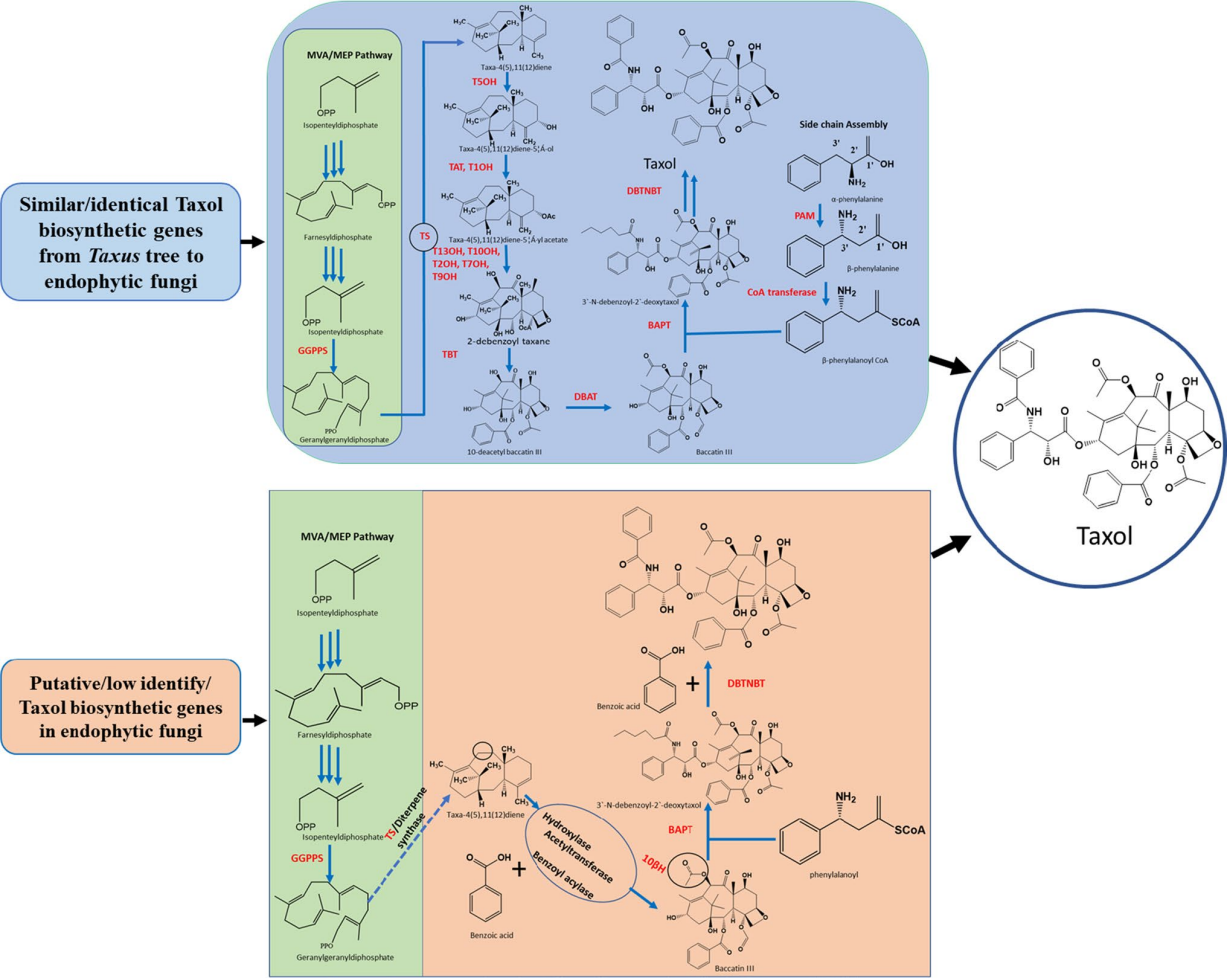
Outline of Taxol biosynthetic pathway in *Taxus* species. Upper image shows that ortholog Taxol biosynthetic pathway genes (Kusari et al. 2014); down image indicated that putative Taxol biosynthetic pathways genes from endophytic fungus (Yang et al. 2014). Abbreviations: BAPT: baccatin III, 3-animo-3-phenylpropanoyltransferase; DBAT, 10-deacetylbaccatin III-10-O-acetyltransferase; DBTNBT, 3′-N-debenzoyl-2′-deoxytaxol-N-benzoyltransferase; GGPPS, geranylgeranyl diphosphate synthase; PAM, phenylalanine aminomutase; TAT, taxadien-5α-ol-O-acetyl transferase; TBT, taxane 2α-O-benzoyltransferase; TS, taxa-4(5),11(12)-diene synthase; T1OH, taxane 1β-hydroxylase; T2OH: taxane 2α-hydroxylase; T5OH, cytochrome P450 taxadiene 5α-hydroxylase;T7OH, taxane 7β-hydroxylase; T10OH, taxane 10β-hydroxylase; T13OH, taxane 13α-hydroxylase. Genes responsible for C1 hydroxylation, oxetane formation, C9 oxidation, β-phenylalanoyl CoA formation, and C2‵hydroxylation of paclitaxel biosynthetic pathway in *Taxus* sp. remain to be elucidated (marked with red color)

*Aspergillus aculeatinus* lacks the key gene for TS found in *Taxus* plants (Qiao et al. 2020). Comparative genomic analyses have long sought to identify paclitaxel pathway genes that are similar to those in the *Taxus* plant (Yang et al. 2014; Miao et al. 2018; Qiao et al. 2020). Nevertheless, genome and transcriptomics data indicated that the fungi may have some putative Taxol biosynthesis genes (Fig. 3). It is possible that the two organisms have evolved different pathways for producing Taxol as a result of the lack of sequence similarity between the endophytic fungi and yew tree. Gibberellin pathways differ between plants and microbes (Yang et al. 2014; Hedden et al. 2001; Soliman et al. 2013). To date, few Taxol producing endophytic fungi have been found for a next-generation sequencing (Table 1). However, little information is available about the putative Taxol biosynthetic genes as follows.

**Table 1:** Analysis of next-generation sequencing for the discovering putative Taxol biosynthetic genes from Taxol producing endophytic fungi

S. no	Taxol producing fungi	Host plant	Sequencing platform	Putative Taxol biosynthetic gene homologs	References
1	*P. aurantiogriseum* NRRL 62431	*Corylus avellana*	WGS (Illumina GA2)	PAM, GGPPS, P450, acetyltransfease	Yang et al. (2014)
2	*C. cladosporioides* MD2	*T. media*	de novo RNA-seq (Illumina HiSeq 2500)	T5αH, T13αH	Miao et al. (2018)
3	*A*. *aculeatinus* Tax-6, BT-2	*T. chinensis var. mairei*	de novo RNA-seq (Illumina HiSeq 2000)	DXR, HDR, HMGS, HMDR, IPPS, GGPPS, T10βH	Qiao et al. (2020)
4	Fungal sp. EF0021	*Taxus* spp*.*	454 GS FLX titanium	Terpene synthease	Cheng et al. (2022)
5	*G. lineata* SDL-CO-2015–1	*Corchorus olitorius*	Illumina HiSeq and PacBio RSII with one SMRT cell	-	Das et al. (2017b)
6	*T. andreanae* CBS 279.92	*T. brevifolia*	Illumina HiSEQ 200	-	Heinig et al. (2013)

### Biosynthesis of isopentenyl pyrophosphate (IPP) and diterpene

Isoprenoids are very important precursors for Taxol biosynthesis in the mevalonate (MVA) and 2-C-methyl-D-erythritol 4-phosphate (MEP) pathways (Bick and Lange 2003). Transcriptomic analysis of the endophytic fungus *A. aculeatinus* Tax-6 reveals that fungal gene such as 1-deoxy-D-xylulose 5-phosphate (DXP), 3-hydroxy-3-methyl-glutaryl-CoA synthase (HMGS), 3-hydroxy-3-methyl-glutaryl-CoA reductase (HMGR), and IPPS play an important role in the MVA pathway (Fig. 3). However, TS does not affect Taxol production from A. *aculeatinus* Tax-6 (Qiao et al. 2020).

GGPP synthase enzyme catalyzes the conversion of three molecules of isopentenyl diphosphate (IPP) and one molecule of dimethylallyl diphosphate (DMAPP) into Taxol (Hefner et al. 1998). Moreover, in *A. aculeatinus* BT-2, GGPPS expression is significantly higher than other genes encoding enzymes DXP, HMGS, and IPPS (Qiao et al. 2020). Based on transcriptomic data, the steps for fungal Taxol biosynthesis are predicted (hydroxylation, acetyltransferases, and benzoyl acylation).

The biosynthesis pathway of phomactins is similar to that of paclitaxel, such as the cyclization of GGPP to form phomactatriene (Sugano et al. 1991; Soliman and Tang 2014). Taxadiene’s structure is interestingly similar to that of phomacta-1(14),3(4),7(8)-triene and phomacta-1(15),3(4),7(8)-triene (Tokiwano et al. 2005; Soliman and Tang 2014). Identifying the biosynthetic genes involved in the production of phomactatriene will certainly lead to the discovery of enzymes related to Taxol biosynthesis pathways in fungi (Soliman and Tang 2014). It is reported that the putative terpene synthase gene sequence is present in the genome of the endophytic fungal EF0021. It is close in structure to the genes for lanosterol synthase, fusicoccadiene synthase, and sesquiterpene synthase (Heinig et al. 2013). Diterpene synthases from *Taxus* species differ in certain ways from EF0021’s putative diterpene synthase gene. Furthermore, the endophyte protein has characteristics of a fungal synthase (diterpene prenyl precursor) and has not been detected in plants. In contrast to the hypothesis that this enzyme is a fungal TS, this may point to its role in diterpene metabolism. However, it has not been proven that the putative terpene synthases gene is involved in fungal Taxol synthesis under various fermentation conditions. A transcriptomic analysis of *C*. *cladosporioides *MD2 revealed that unigene c11040_g1 was homologous to a taxadiene synthase gene (Miao et al. 2018).

### Hydroxylase and other enzymes

According to Kyoto encyclopedia of genes and genome analysis of transcriptomic data, *C. cladosporioides* MD2 has a fungal Taxol biosynthesis pathway. This observation is supported by the identification of unigenes that show homology to the T5αH and T13αH genes, which are known to be involved T13αH, which catalyzed the hydroxylation of taxane C13 in Taxol biosynthesis (Miao et al. 2018; Jennewein et al. 2001). Nevertheless, it might be fungal in origin and needed for the biosynthesis of 10-deacetyl-2-debenzoylbaccatin III. *Taxus*-derived Taxol is synthesized by a diterpene olefin precursor hydroxylation by cytochrome p450 (Wheeler et al. 2001; Jennewein et al. 2004). This is followed by a fungal cytochrome 450 from *P. aurantiogriseum* NRRL 62431 which exhibited 48% identity with *Taxus* plants (Yang et al. 2014). Cytochrome 450 oxidoreductase involved in aflatoxin formation in *Aspergillus* has been reported to perform different reactions such as hydroxylation, desaturation, or oxidation (Payne and Brown 1998; Črešnar and Petrič 2011).

It is the first step in the side chain biosynthesis of Taxol to convert alpha-phenylalanine into beta-phenylalanine using PAM. *P. aurantiogriseum* NRRL 62431, an endophytic fungus isolated from *Taxus* species, is capable of synthesizing paclitaxel, based on its genome sequence. According to Yang et al. (2014), the conserved amino acid sequence of the paclitaxel biosynthetic candidate PAM genes has 43% identity with its ortholog in the *Taxus* plant. A complete study of the enzymes and genes involved in the Taxol biosynthesis pathway has not yet been conducted.

### Acetyltransferase and benzoyltransferase

A gene coding for acetyltransferase in *P. aurantiogriseum* has been found in different clades of *Taxus* plants, according to Yang et al. (2014). As a result, these findings suggest that the acetyltransferase genes from *Taxus* and *P. aurantiogriseum* differ significantly in genetic diversity and evolutionary divergence. In the Taxol biosynthetic pathway, the unigene c5600_g1 catalyzed the conversion of a 2-debenzoyl intermediate, called a taxoid-type intermediate, to 10-deacetylbaccatin III (10-DAB), a late-stage acylation step (Walker and Croteau 2000a, b; Miao et al. 2018).

## Taxol-producing endophytic fungi with the highest yield

Over 200 endophytic fungi are found in several orders, including Ascomycota, Deuteromycetes, Coelomycetes, Basidiomycota, and Zygomycota, among others. Several studies have reported the production of Taxol (McElroy and Jennewein 2018; Gond et al. 2014; Kusari et al. 2014; Flores-Bustamante et al. 2010; Naik 2019; Tasiu 2015). It has been reported that all endophytic fungi produce Taxol under laboratory conditions in the absence of *Taxus* plants. Several early and recent studies investigated Taxol production by thin-layer chromatography, high-performance liquid chromatography, liquid chromatographic–electrospray ionization tandem mass-spectrometric, and nuclear magnetic resonance (Zhou et al. 2010; Naik 2019; Gond et al. 2014; El-Sayed et al. 2019a, b, 2020; Chakravarthi et al. 2008, 2020; Guo et al 2006). A few studies used competitive inhibition enzyme immune assays (Chakravarthi et al. 2020; Heinig et al. 2013), which may interfere with other toxoid-like molecules. Therefore, these immunological methods have led to the misidentification of Taxol (McElroy and Jennewein 2018).

Even closely related strains can produce different levels of Taxol: Stierle et al. (1993) reported a low yield of 24–25 ng/L for *Pestalotiopsis hainanensis*, while Gu et al. (2015) reported a high yield (1.46 mg/L). In light of this, more attention must be paid in the future to finding suitable endophytic fungal strains. Table 2 shows only a few fungi that produce Taxol between 500 µg and 1 mg/L of culture.

**Table 2:** List of the highest (more than 500 µg/L) Taxol-producing endophytic fungi and their host plants

S. no	Endophytic fungi	Plant host	Taxol yield (µg/L)	References
1	*Metarrhizium anisopliae* H-27	*T.chinensis*	846.1	Liu et al. (2009)
2	*Phoma betae* SBU-16	*Gingko biloba*	795	Kumaran et al. (2012)
3	*C. cladosporioides*	*T. media*	800	Zhang et al. (2009b)
4	*Aspergillus fumigatus* EPTP-1	*Podocarpus* sp.	560	Sun et al. (2008)
5	*Aspergillus fumigatus*	*Taxus* sp.	1590	Kumar et al. (2019)
6	*Cladosporium oxysporum*	*Moringa oleifera*	550	Gokul et al. (2015)
7	*Aspergillus aculeatinus*	*T. chinensis* var*. mairei*	1137.56	Qiao et al. (2017)
8	*Pestalotiopsis hainanensis*	*Ailuropoda melanoleuca*	1466	Gu et al. (2015)

## Taxol derivatives produced by endophytic fungi

**Fig. 4:**
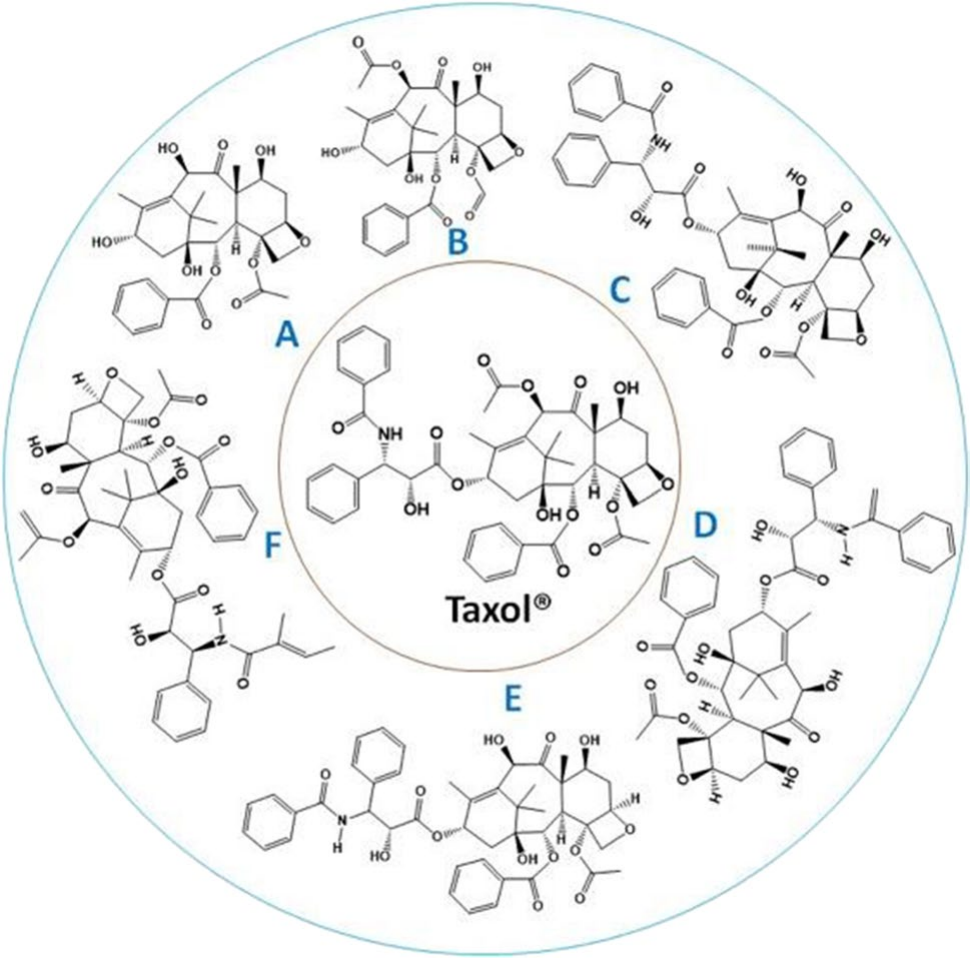
Chemical structure of Taxol and their intermediate products obtained from endophytic fungi: 10-Deacetylbaccatin III (**A**); Baccatin III (**B**); 7-epi-taxol (**C**); 10-Deacetyltaxol (**D**); 7-epi-10-Deacetyltaxol (**E**): Cephalomannine (**F**)

Taxanes can be extracted from both filtrate and fungal residues from dichloromethane organic solution evaporated to dryness and dissolved in methanol. The fungal residue was analyzed by chromatography and spectroscopy. Some Taxol intermediate products of taxanes such as 10 deacetyl baccatin III (Li et al. 2015, 2017; Sreekanth et al. 2009; Chakravarthi et al. 2020; Ballakuti et al. 2022), baccatin III (Chakravarthi et al. 2008, 2020, 2013; Li et al. 2015; Jian et al. 2013; Ballakuti et al. 2022), Cephalomannine (Li et al. 2017; Ballakuti et al. 2022), 7-epi-10 deacetyl Taxol (Ballakuti et al. 2022; Kamalraj et al. 2017), and 7-epi-Taxol (Ballakuti et al. 2022) were reported from endophytic fungi (Fig. 4). Moreover, fungal Taxol intermediate products can be used to manufacture Taxol/taxotere by semi-synthesis. Furthermore, it provides valuable clues to understanding Taxol biosynthesis from endophytic fungi.

## Induction of elicitors

It is generally understood that elicitors work either positively or negatively to regulate secondary metabolic biosynthesis, which determines the expression of genes involved in biosynthetic pathways (Qari and Tarbiyyah 2021). They may also activate or repress defense mechanisms or symbiosis mechanisms in plants and fungi. Due to their heterotrophic nature, endophytic fungi are highly likely to be affected by elicitors. In endophytic fungal cultures, jasmonic acid, salicylic acid, ammonium acetate, sodium acetate, silver nitrate, phenylalanine, serine, sodium acetate, ammonium nitrate, and magnesium sulfate have been shown to elicit Taxol production (Zhao et al. 2011; Somjaipeng et al. 2016; Qiao et al. 2017). *Paraconiothyrium* sp. co-cultivation with *Phomopsis* sp. and *Alternaria* sp. improved Taxol production by eightfold (Soliman and Raizada 2013). A biotic elicitor, *Taxus* bark extract added to a *Paraconiothyrium* SSM001 culture, enhanced fungal Taxol production at 30-fold (Soliman and Raizada 2013). The suspension culture of *Taxus* spp. with *Fusarium* sp. also showed promising results, and the production of Taxol was increased by 38-fold (Li et al. 2009). It indicates that Taxol is produced by the endophyte fungus in conjunction with a native host. Fungal and bacterial co-cultivation seems to have increased expression of certain proteins in *Aspergillus flavipes*, including histones H2B and H2A, peptidyl-prolyl cis–trans isomerase, and nucleoside-diphosphate kinase. These proteins may be involved in chromatin remodeling and the regulation of gene expression, including those related to fungal Taxol biosynthesis (El-Sayed et al. 2021). The increased expression of specific proteins suggests that the chromatin structure in fungus is being altered, which can lead to changes in the accessibility of gene clusters and the activation of silent Taxol biosynthetic pathways (Mohamed et al. 2023). Moreover, as a result of various elicitations and stress factors, fungal Taxol production was significantly increased at least in some fungi (Soliman et al. 2013). In *Pestalotiopsis microspora* cultures, salicylic acid was a powerful elicitor that promoted Taxol production as well as the expression of a putative *ggpp* gene and a specific mycelia protein profile correlated with Taxol production (Kamalraj et al. 2019).

## Precursor feeding

In order to increase Taxol production, suitable precursors are fed into the fungal culture medium (Stierle et al. 1995). The main issue limiting Taxol biosynthesis is long storage and frequent subculturing of fungi. In this context of restoring and enhancing the Taxol biosynthetic potency by epigenetic regulating mechanisms such as epigenetic regulators, transcriptional factors, metabolic manipulators, microbial communications, and microbial cross-talking in endophytic fungi is of great relevance (Mohamed et al. 2023). Using reverse transcription-quantitative polymerase chain reaction (RT-qPCR), the expression of Taxol biosynthesis rate-limiting genes, such as *ts*, *dbat*, and *bapt*, was confirmed in an *Aspergillus* isolate with influx of acetyl-CoA (El-Sayed et al. 2019b). The presence of sufficient acetyl-CoA in a microbial cell also facilitates the production of baccatin III (Huang et al. 2022). The combination of p-hydroxybenzoic acid, serinol, and phenolic acids from *Periconia* sp. induced phenylpropanoid metabolism which regulates Taxol biosynthesis (Li et al. 1998b). A recent study shows that PAL enzyme activity was induced with benzoic acid as an elicitor in the endophytic fungus *Cryptosporiopsis tarraconensis* (Ballakuti and Ghanati 2023). The aminomutase catalyzes the transformation of α-phenylalanine into β-phenylalanine, which is further transformed into phenylazirine and followed by hydroxylating at the C2 site (Zhao et al. 2016). Jiang et al. (2012) describe phenylisoserine as an important precursor of Taxol side chain formation. Thus, acetate and phenylalanine are important precursors of fungal Taxol biosynthesis (Stierle et al. 1993). In sum, these studies indicate that terpenoid or phenylpropanoid pathways are most likely to serve as targets for future improvements in Taxol biosynthesis.

## Use of inhibitors to identify Taxol biosynthetic pathway genes and enzymes

The importance of the mevalonate and non-mevalonate pathway in fungal paclitaxel biosynthesis was shown by adding specific inhibitors, i.e., compactin and fosmidomycin, which inhibit the 3-hydroxy-3-methyl-glutaryl-CoA reductase enzyme and 1-deoxy-d-xylulose-5-phosphate reductoisomerase (DXR), respectively (Soliman et al. 2011). Based on the inhibition of DXR (non-MVA pathway), fungi may require the DXR enzyme to form a MEP pathway similar to plants and bacteria. Further evidence comes from methyl jasmonate and benzoic acid treated fungal cultures of *C. tarraconensis* showing increased DXR enzyme activity and production of DAB and baccatin III (Ballakuti and Ghanati 2023). In Taxol producing fungi, the presence of precursors such as acetate and phenylalanine increased sterol biosynthesis, while tebuconazole and triadimefon inhibited sterol biosynthesis (Li et al. 1998a; El-Sayed et al. 2019a, b). In addition to fluconazole, which correlates with Taxol yield and Taxol biosynthesis, genes *ts*, *dbat*, and *bapt* were significantly upregulated in *Aspergillus*. In those Taxol biosynthetic genes, however, the conserved gene sequence does not exist, so the effect is only temporary.

## Conclusion and future prospects

In this review, we demonstrate that endophytic fungi can produce Taxol regardless of their host species. Fungal Taxol production may be genetically derived from HGT from the host plant to its endophytes or from convergent evolution. Further investigation is hindered by limited information on Taxol biosynthetic orthologs from Taxol producing fungi. Alternatively, fungi could have evolved an independent Taxol biosynthesis system after losing their ortholog genes. Genomic sequence analysis, transcriptomic data, and homology searches are often used in the prediction of genes involved in the Taxol biosynthetic pathway. For putative taxol biosynthesis genes to be confirmed, further verification and characterization are required. When all the biosynthetic steps are fully characterized, large-scale production of the fungal Taxol will be possible. Fermentation technology, genetic engineering, synthetic biology, and metabolic engineering approaches offer a possible way to counteract fungal Taxol production and provide a remarkable benefit to society.
